# Changes and continuity in the editorial management of História, Ciências, Saúde – Manguinhos in 2026

**DOI:** 10.1590/S0104-59702026000100008en

**Published:** 2026-05-11

**Authors:** Marcos Cueto, Gabriel Lopes

**Affiliations:** i Editor-in-chief, researcher, Casa de Oswaldo Cruz/Fiocruz. Rio de Janeiro – RJ – Brazil; ii Editor-in-chief, researcher, Casa de Oswaldo Cruz/Fiocruz. Rio de Janeiro – RJ – Brazil

On January 1, 2026 an important transition occurred in the editorship of *História, Ciências, Saúde – Manguinhos*, preceded by changes in the team of assistant editors several months earlier. By means of this letter, we (Marcos Cueto and Gabriel Lopes) wish to jointly notify readers of this development.

Current Editor-in-Chief Marcos Cueto, who assumed this post in 2015 after Jaime L. Benchimol and shared it with André Felipe Cândido da Silva for a five-year period, will be stepping down. This past ten years of work has involved landmark events dedicated to discussing the tribulations of journal publishing in Brazil, Latin America and other regions of the world, as well as disseminating our materials on the journal’s blog and social networks and preparing proposals for domestic and international funding. Highlights include support from the Brazilian National Council for Scientific and Technological Development and the Wellcome Foundation in the past. Above all, this was a period of constant learning marked by work on notable supplements, as well as manuscripts and opinions that explored the frontiers of historical knowledge, both expanding and refining the historiographical debate on various topics. One custom we adopted years ago was to use the first Editor’s Note of the year to summarize progress made as well as challenges facing the journal, a habit that will certainly continue into this new phase (Cueto, 2025; Cerqueira, Cueto, 2024; Cueto, 2023). These letters record crucial moments in the journal’s history and its active participation in organizing fundamental debates, such as threats to democracy and the challenges in the field of global health during the covid-19 pandemic.

Last year marked the start of a fundamental task led by Roberta Cardoso Cerqueira, executive editor of *História, Ciências, Saúde – Manguinhos*: to ensure and expand the journal’s presence in indexing services beyond the better-known platforms such as SciELO, Redalyc, Scopus and PubMed. These efforts provide more credibility and accessibility for published articles, standardize good editorial practices and offer researchers consistent parameters related to the relevance and reliability of the journal. During this expansion and under the guidance of Marcos Cueto and Roberta Cardoso Cerqueira, Gabriel Lopes underwent extensive training as co-editor (following his appointment as a researcher at Casa de Oswaldo Cruz in the final months of 2025), observing all stages in the editorial process and scientific dissemination work on the blog and social networks of *História, Ciências, Saúde – Manguinhos*, helping to jointly define the journal’s next steps and priorities from 2026 onwards (Lopes, ago. 2025).

Marcos Cueto’s farewell as editor-in-chief is accompanied by his deep gratitude to the journal’s extraordinary team, led by Roberta Cardoso Cerqueira, to the assistant and section editors, to the members of the editorial board, authors, reviewers and readers, as well as to the Board of Casa de Oswaldo Cruz and to Abel Parker, director of SciELO. The new management, under the leadership of Gabriel Lopes, is committed to continuing this legacy of professionalism and internationalization. While Gabriel takes over as editor-in-chief, Marcos Cueto will continue as a researcher at Casa de Oswaldo Cruz and professor in the Graduate Program in the History of Science and Health (PPGHCS).

As part of his responsibility for the new editorial management, Gabriel Lopes will seek to reinforce the scientific quality and ethical integrity of the journal, facing new challenges (such as those posed by artificial intelligence) and following the editorial process in the continuous publication model we adopted over two years ago. He will also dedicate himself to incorporating appropriate innovations into editorial policy and the instructions to authors when necessary, strengthening the journal’s commitment to open science and expanding its capacity to attract original, relevant, high-quality contributions from Brazil and abroad that are in line with contemporary debates in the history of science and health.

Although responsibilities change, our commitment to the quality of a journal that is heading towards 35 years of existence remains unchanged – a remarkable achievement in a country in the Global South. Working closely with Gabriel Lopes, Marcos Cueto will continue to provide support over several months for specific activities at the journal related to reviews of manuscripts submitted during the second half of 2025 with final decisions that are expected to conclude in the first months of 2026. Alongside Gabriel Lopes and Roberta Cardoso Cerqueira, he will also participate in organizing a workshop for PPGHCS students entitled “Challenges of scientific publication in open science and history journals,” and will also continue to contribute in activities to internationalize *História, Ciências, Saúde – Manguinhos*.

The journal continues on its strengthened trajectory with the commitment of its entire editorial team and the academic community of researchers, reviewers and authors who support it. Far from representing a rupture, this transition highlights the institutional vitality and shared commitment to scientific excellence that have characterized the journal for more than three decades. Under the leadership of Gabriel Lopes as editor-in-chief, and thanks to the well-established work of Roberta Cardoso Cerqueira as executive-editor, *História, Ciências, Saúde – Manguinhos* reaffirms its vocation to publishing high-quality research on the history of science and health and to opening the journal up to new topics such as animal history, the Anthropocene, and health and the environment. Recently, the journal was once again recognized in the scientific journal evaluation by the Coordination for the Improvement of Higher Education Personnel (2021-2024), maintaining the highest rating (A1 qualification), reaffirming its relevance and leadership in the field. Its future path points toward expanding its international reach and contributing to contemporary debates in the field, in line with the consolidation of open science in defense of knowledge as a public asset.

Many challenges lie ahead, but the changes that are already being implemented, together with accumulated experience and recognition of everyone’s efforts, inspire confidence that *História, Ciências, Saúde – Manguinhos* will continue to be a fundamental reference in the production and dissemination of historical knowledge.
