# Supplementary material for: Changes and continuity in the editorial management of História, Ciências, Saúde – Manguinhos in 2026
Source: Hist Cienc Saude Manguinhos. 2026 May 11;33:e2026008. doi: 10.1590/S0104-59702026000100008en (PMC13170110; doi:10.1590/S0104-59702026000100008en)
Supplement: Translation

# Translation: Mudança e continuidade na gestão editorial de História, Ciências, Saúde – Manguinhos em 2026

**DOI:** 10.1590/S0104-59702026000100008

**Authors:** Marcos Cueto, Gabriel Lopes

**Affiliations:** i Editor-chefe, pesquisador, Casa de Oswaldo Cruz/Fiocruz. Rio de Janeiro – RJ – Brasil; ii Editor-chefe, pesquisador, Casa de Oswaldo Cruz/Fiocruz. Rio de Janeiro – RJ – Brasil

A partir de 1º de janeiro de 2026 ocorreu uma importante transição na editoria de *História, Ciências, Saúde – Manguinhos*, precedida por mudanças recentes na equipe de editores adjuntos realizadas alguns meses antes. Por meio desta carta, nós – Marcos Cueto e Gabriel Lopes – desejamos comunicar conjuntamente essa mudança.

Marcos Cueto, atual editor-chefe, que recebeu o cargo de Jaime L. Benchimol em 2015 e o compartilhou com André Felipe Cândido da Silva nos cinco primeiros anos, deixará essa função. Foram dez anos de trabalho, com eventos marcantes dedicados a discutir as atribulações da editoria de revistas no Brasil, na América Latina e em outras regiões do mundo; anos que também envolveram a difusão de nossos materiais no *blog* da revista e nas redes sociais, bem como a preparação de propostas para financiamentos nacionais e internacionais – dentre os quais se destacam o apoio recebido do Conselho Nacional de Desenvolvimento Científico e Tecnológico e, anos atrás, da Fundação Wellcome. Sobretudo, foi um período de aprendizado constante, marcado pelo trabalho com suplementos notáveis, assim como manuscritos e pareceres que exploraram a fronteira do conhecimento histórico não apenas expandindo, mas refinando o debate historiográfico em diversos temas. Um costume que adotamos nos últimos anos foi o de resumir os avanços alcançados e os desafios da revista na primeira carta do editor de cada ano, um hábito que certamente continuará nesta nova fase da revista (Cueto, 2025; Cerqueira, Cueto, 2024; Cueto, 2023). Essas cartas registram momentos cruciais da história da revista e sua participação ativa na organização de debates fundamentais, como ameaças antidemocráticas e os desafios no campo da saúde global no decorrer da pandemia de covid-19.

Em 2025, iniciou-se uma tarefa fundamental, liderada por Roberta Cardoso Cerqueira, editora executiva da revista: garantir e ampliar a presença do periódico em indexadores – além dos mais conhecidos, como SciELO, Redalyc, Scopus e PubMed –, conferindo mais credibilidade e acessibilidade aos artigos publicados, padronizando boas práticas editoriais e oferecendo aos pesquisadores parâmetros consistentes de relevância e confiabilidade de *História, Ciências, Saúde – Manguinhos*. Durante essa ampliação, sob a orientação de Marcos Cueto e Roberta Cardoso Cerqueira, Gabriel Lopes passou por intenso treinamento como coeditor – após sua nomeação como pesquisador da Casa de Oswaldo Cruz nos últimos meses de 2025 –, acompanhando todas as fases do processo editorial e do trabalho de divulgação científica no *blog* da revista e nas redes sociais, ajudando a definir, em conjunto, os próximos passos e prioridades da revista a partir de 2026 (Lopes, ago. 2025).

A despedida de Marcos Cueto da editoria é acompanhada de profunda gratidão à extraordinária equipe da revista, liderada por Roberta Cardoso Cerqueira, aos editores- adjuntos e de seção, aos membros do corpo editorial, autores, avaliadores e leitores, assim como à Direção da Casa de Oswaldo Cruz e a Abel Parker, diretor da SciELO. A nova gestão, sob a liderança de Gabriel Lopes, herda o compromisso de dar continuidade a esse legado de profissionalismo e de internacionalização. Enquanto Gabriel assume a editoria-chefe, Marcos Cueto seguirá como pesquisador da Casa de Oswaldo Cruz e professor do Programa de Pós-graduação em História das Ciências e da Saúde (PPGHCS).

Gabriel Lopes, responsável pela nova gestão editorial, buscará reforçar a qualidade científica e a integridade ética da revista, enfrentando novos desafios – como aqueles colocados pela inteligência artificial – e acompanhando o fluxo editorial em nosso modelo de publicação contínua, adotado há mais de dois anos. Também se dedicará a incorporar inovações adequadas à linha editorial e às instruções aos autores quando necessário, fortalecer o compromisso com a ciência aberta e ampliar a capacidade da revista de atrair contribuições nacionais e internacionais originais, relevantes, de alta qualidade e alinhadas aos debates contemporâneos na história das ciências e da saúde.

Embora as responsabilidades se modifiquem, permanece inalterado nosso compromisso com a qualidade de uma revista que se encaminha para seus 35 anos de existência – um feito notável em um país do Sul Global. Durante alguns meses Marcos Cueto continuará apoiando atividades pontuais da revista, em estreita cooperação com Gabriel Lopes, no que se refere aos pareceres de manuscritos encaminhados no segundo semestre de 2025 e cuja decisão final deverá ser concluída nos primeiros meses de 2026. Também participará, ao lado de Gabriel Lopes e Roberta Cardoso Cerqueira, da organização da oficina “Desafios da publicação científica nos periódicos de história e a ciência aberta”, dirigida aos alunos do PPGHCS, bem como seguirá contribuindo com as ações de internacionalização da revista.

A revista prossegue sua trajetória solidificada pelo empenho de toda sua equipe editorial e da comunidade acadêmica de pesquisadores, avaliadores e autores que a sustentam. Essa transição, longe de representar uma ruptura, evidencia a vitalidade institucional e o compromisso compartilhado com a excelência científica que caracterizam o periódico há mais de três décadas. Sob a liderança de Gabriel Lopes, como editor-chefe, e o trabalho já consolidado de Roberta Cardoso Cerqueira na editoria executiva, *História, Ciências, Saúde – Manguinhos* reafirma sua vocação de publicar pesquisas de qualidade em história das ciências e da saúde e abre o periódico para novas temáticas como a histórias dos animais, o Antropoceno, e saúde e meio ambiente. Recentemente, a revista foi mais uma vez reconhecida na avaliação de periódicos científicos da Coordenação de Aperfeiçoamento de Pessoal de Nível Superior (2021-2024), mantendo o conceito máximo (qualificação A1), reafirmando sua relevância e sua liderança na área. Seu caminho futuro aponta para a ampliação do alcance internacional e da contribuição aos debates contemporâneos do campo, alinhando-se à consolidação da ciência aberta na defesa do conhecimento como bem público.

As atribulações não são menores, e novos desafios surgem, mas a renovação em curso, realizada em sintonia com a experiência acumulada e com o reconhecimento do esforço de todos, inspira a confiança de que *História, Ciências, Saúde – Manguinhos* continuará seguindo como referência fundamental na produção e divulgação do conhecimento histórico.
